# Editorial: Advances in genomics, genetics, and breeding of the cucurbit plant

**DOI:** 10.3389/fpls.2024.1378952

**Published:** 2024-03-07

**Authors:** Shenglin Wang, Weiwei Zhang, Jingtao Nie

**Affiliations:** ^1^College of Horticulture Science, Zhejiang Agriculture & Forestry University, Hangzhou, Zhejiang, China; ^2^Department of Plant Science and Technology, Shanghai Vocational College of Agriculture and Forestry, Shanghai, China; ^3^Key Laboratory of Quality and Safety Control for Subtropical Fruit and Vegetable, Ministry of Agriculture and Rural Affairs, Hangzhou, Zhejiang, China; ^4^Collaborative Innovation Center for Efficient and Green Production of Agriculture in Mountainous Areas of Zhejiang Province, College of Horticulture Science, Zhejiang Agriculture & Forestry University, Hangzhou, Zhejiang, China

**Keywords:** cucurbitaceae, genomics, genetics, breeding, advance

Cucurbits, the second largest vegetable family, encompass a wide variety of vegetables such as watermelon, melon, pumpkin, cucumber, bitter melon, bitter gourd, wax gourd, and numerous other species. The importance of cucurbit crops as a food source is recognized worldwide, and researchers have consistently focused on improving their quality and stress resistance. Firstly, as people’s living standards improve, there is a growing demand for high-quality cucurbits, which in turn requires the cultivation of new and improved germplasms. Secondly, the detrimental effects of various biotic and abiotic stresses significantly impact the yield and food safety of cucurbit crops. Therefore, studying the stress resistance of cucurbit crops is crucial for ensuring safe and efficient production. By investigating the traits related to quality and stress resistance in cucurbit crops, valuable theoretical support can be provided for the development of innovative germplasms.

In recent years, significant progress has been made in the genomics of cucurbit crops, including cucumber, melon, and watermelon (http://cucurbitgenomics.org/). This has led to rapid development in the genetics of these crops. High-density genetic maps have been established, and there has been great progress in map-based cloning of desirable traits. Additionally, the completion of genome sequencing has facilitated molecular biology research using omics methods such as genome sequencing and transcriptome sequencing (http://cucurbitgenomics.org/). These advancements have provided convenience for studying the genetics and molecular biology of agronomic traits in cucurbit crops, ultimately benefiting their genetic breeding. The purpose of this topic is to highlight the progress made in genomics, genetics, and breeding of cucurbit crops, with the aim of establishing a solid foundation for breeding desirable traits in these crops.

Several important traits controlled by a single gene were genetically mapped for cucurbit crops and candidate genes were proposed. Niu et al. showed that the yellow peel trait in zucchini (*Cucurbita pepo*) was controlled by a single dominant gene Y and mapped in a ~170 kb region on chromosome 10 by bulked segregated analysis (BSA) and fine mapping in segregating populations F_2_ and BC_1_. Fifteen annotated genes existed in this region and *Cp4.1LG10g11560* (*CpCHLH*) was considered a promising candidate gene. A large fragment (~15 kb) containing incomplete *CpCHLH* was inserted into the candidate interval, resulting in two reformed CpCHLH proteins in the yellow parental line. Gebremeskel et al. showed that the delayed green leaf color in watermelon (*Citrullus lanatus*) was dominated by a single recessive gene. The delayed green (*dg*) locus was mapped to 7.48 Mb on chromosome 3 using the BSA approach and then narrowed to a 53.54 kb region containing three candidate genes. A single SNP variation in *ClCG03G010030* can result in early delayed green leaf color development and the expression level of *ClCG03G010030* was significantly reduced in delayed green leaf plants than in green leaf plants.

In addition, several important traits controlled by multiple loci were also genetically mapped by QTL (quantitative trait loci) mapping for cucurbit crops. Lin et al. identified QTLs associated with resistance to phytophthora fruit rot (PFR) using multiple genomic approaches and populations. Two types of resistance have been identified: age-related resistance (ARR) and young fruit resistance. A major QTL for ARR was discovered on chromosome 3 and a candidate gene was identified on the basis of comparative transcriptomic analysis. For resistance of young fruits, multiple QTLs were identified on chromosomes 1, 5, and 6, and the most significant QTL, qPFR5.1, located on chromosome 5 was fine mapped to a region of 1 Mb. Genome-wide association studies (GWAS) were also performed for resistance of young fruits. Several SNPs overlapped with the QTL identified from QTL-seq analysis, and novel SNPs associated with resistance were also identified. Wang et al. performed QTL mapping for trichome density in zucchini (*Cucurbita pepo* L.) using both QTL-seq and genetic map-based QTL analyses. Two QTLs, *CpTD3.1* and *CpTD15.1*, were identified in both methods, and *CpTD15.1* was identified as the QTL with the largest effect. *CpTD15.1* was located at a physical distance of 775.44 kb and explained 12.71% to 29.37% of the phenotypic variation in the three environments. The functional annotations of the genes within the *CpTD15.1* region were predicted and *Cp4.1LG15g04400*, which encodes a zinc finger protein (ZFP), was presumed to be the candidate gene to regulate the density of the trichome in zucchini. Xing et al. constructed a genetic map containing 600 bin markers via re-sequencing and then performed QTL mapping and transcriptomic analysis for fruit length in cucumber. It revealed three QTLs (*Fl2.1*, *Fl4.1*, and *Fl6.1*) repeatedly detected in the two seasons, of which *Fl4.1* was the major QTL. The *Csa4G337340* gene encoding an auxin efflux carrier family protein appeared as the candidate gene by expression analysis. Additionally, genes related to plant hormone signal transduction and several transcription factors were also found to be involved in the regulation of cucumber fruit length through transcriptomic analysis.

Several genes and pathways related to adventitious root (AR) formation were characterized. Li et al. investigated the mechanisms of strigolactone and auxin in AR formation in melon, and auxin promotes strigolactone-induced adventitious root growth in the hypocotyl of melon seedlings. Transcriptome analysis revealed that this mechanism was achieved by affecting the expression of genes related to the pathways and contents of plant hormones.

Genome-wide gene family characterization was also performed. Yuan et al. identified the *CsABCG* gene family in cucumber. Phylogenetic analysis, sequence alignment, and collinear analysis indicated that the functions of ABCG proteins in different plants are evolutionarily conserved. The cis-acting elements and expression analysis showed that they played an important role in the development and response to various biotic and abiotic stresses in cucumbers. In addition, potential binding sites for the miRNA-targeted *CsABCG* genes were also predicted.

A review was conducted to summarize the progress of sex differentiation for cucurbit crops. Luo et al. reviewed the research progress on sex differentiation in cucumber in recent years, focusing mainly on sex-determining genes, environmental conditions, and the influence of phytohormones in cucumber, and provides a theoretical basis and technical support for the realization of high and stable yield cultivation and molecular breeding of cucumber crop traits.

Although cucurbit crops have recently achieved promising results in the fields of genomics and genetic breeding, many problems need to be solved urgently. For example, although the genes controlling certain traits have been cloned, their intrinsic molecular mechanisms and regulatory networks have not yet been clearly analyzed; some important traits are controlled by QTLs and are greatly affected by the environment, making map-based cloning of target genes difficult; the cloning and mechanism research of target genes for some important traits, such as disease resistance and stress resistance, are progressing slowly because they involve the interaction between microorganisms, environment and the crops, there are many influencing factors, resulting in a relatively lagging research process. These will hinder the application and promotion of molecular breeding in cucurbit crops. Even so, scientists in related fields are already working hard to overcome these difficulties. We are already on the way and believe that in the near future, we will bring rapid progress to the genetic breeding of cucurbit crops.

Finally, as Guest Editors, we would like to thank all the authors and co-authors for their valuable contributions to this Research Topic, and all the reviewers for their important work in evaluating the submitted manuscripts. We expect This Research Topic to contribute to the advancement of genetic improvement for cucurbit crops and believe it serves as a valuable reference to our global colleagues.

## Author contributions

JN: Writing – original draft, Writing – review & editing. WZ: Writing – review & editing. SW: Writing – original draft.

